# Semaglutide promotes bone marrow–derived progenitor cell flux towards an anti-inflammatory and pro-regenerative profile in high-risk patients: the SEMA-VR CardioLink-15 trial

**DOI:** 10.1093/eurheartj/ehaf690

**Published:** 2025-08-31

**Authors:** Brady Park, Fallon Dennis, Arianna Z He, Aishwarya Krishnaraj, Ehab Bakbak, Cole J Dennis, Yi Pan, Elizabeth Misner, Veena Thayanithy, Bhavaani Lambotharan, Vaasudevan Lambotharan, Aruna Lambotharan, C David Mazer, Adrian Quan, Hwee Teoh, David A Hess, Subodh Verma

**Affiliations:** Division of Cardiac Surgery, St. Michael’s Hospital of Unity Health Toronto, 30 Bond Street, Toronto M5B 1W8, ON, Canada; Keenan Research Centre of Biomedical Science and Li Ka Shing Knowledge Institute, St. Michael’s Hospital of Unity Health Toronto, 209 Victoria Street, Toronto M5B 1X3, ON, Canada; Department of Pharmacology and Toxicology, University of Toronto, 1 King's College Circle, Toronto M5S 3K3, ON, Canada; Division of Cardiac Surgery, St. Michael’s Hospital of Unity Health Toronto, 30 Bond Street, Toronto M5B 1W8, ON, Canada; Keenan Research Centre of Biomedical Science and Li Ka Shing Knowledge Institute, St. Michael’s Hospital of Unity Health Toronto, 209 Victoria Street, Toronto M5B 1X3, ON, Canada; Department of Pharmacology and Toxicology, University of Toronto, 1 King's College Circle, Toronto M5S 3K3, ON, Canada; Division of Cardiac Surgery, St. Michael’s Hospital of Unity Health Toronto, 30 Bond Street, Toronto M5B 1W8, ON, Canada; Keenan Research Centre of Biomedical Science and Li Ka Shing Knowledge Institute, St. Michael’s Hospital of Unity Health Toronto, 209 Victoria Street, Toronto M5B 1X3, ON, Canada; Department of Pharmacology and Toxicology, University of Toronto, 1 King's College Circle, Toronto M5S 3K3, ON, Canada; Division of Cardiac Surgery, St. Michael’s Hospital of Unity Health Toronto, 30 Bond Street, Toronto M5B 1W8, ON, Canada; Keenan Research Centre of Biomedical Science and Li Ka Shing Knowledge Institute, St. Michael’s Hospital of Unity Health Toronto, 209 Victoria Street, Toronto M5B 1X3, ON, Canada; Department of Pharmacology and Toxicology, University of Toronto, 1 King's College Circle, Toronto M5S 3K3, ON, Canada; Faculty of Medicine, University of Queensland, Brisbane, Australia; Division of Cardiac Surgery, St. Michael’s Hospital of Unity Health Toronto, 30 Bond Street, Toronto M5B 1W8, ON, Canada; Keenan Research Centre of Biomedical Science and Li Ka Shing Knowledge Institute, St. Michael’s Hospital of Unity Health Toronto, 209 Victoria Street, Toronto M5B 1X3, ON, Canada; Department of Pharmacology and Toxicology, University of Toronto, 1 King's College Circle, Toronto M5S 3K3, ON, Canada; Division of Cardiac Surgery, St. Michael’s Hospital of Unity Health Toronto, 30 Bond Street, Toronto M5B 1W8, ON, Canada; Keenan Research Centre of Biomedical Science and Li Ka Shing Knowledge Institute, St. Michael’s Hospital of Unity Health Toronto, 209 Victoria Street, Toronto M5B 1X3, ON, Canada; Division of Cardiac Surgery, St. Michael’s Hospital of Unity Health Toronto, 30 Bond Street, Toronto M5B 1W8, ON, Canada; North York Diagnostic and Cardiac Centre, Toronto, ON, Canada; Legacy Medical Centre, Toronto, ON, Canada; Legacy Medical Centre, Toronto, ON, Canada; Legacy Medical Centre, Toronto, ON, Canada; North West of England School of Foundation Training and Physician Associates, Manchester, United Kingdom; Legacy Medical Centre, Toronto, ON, Canada; Keenan Research Centre of Biomedical Science and Li Ka Shing Knowledge Institute, St. Michael’s Hospital of Unity Health Toronto, 209 Victoria Street, Toronto M5B 1X3, ON, Canada; Department of Pharmacology and Toxicology, University of Toronto, 1 King's College Circle, Toronto M5S 3K3, ON, Canada; Department of Anesthesia, St. Michael’s Hospital of Unity Health Toronto, Toronto, ON, Canada; Department of Anesthesiology and Pain Medicine, University of Toronto, Toronto, ON, Canada; Department of Physiology, University of Toronto, Toronto, ON, Canada; Division of Cardiac Surgery, St. Michael’s Hospital of Unity Health Toronto, 30 Bond Street, Toronto M5B 1W8, ON, Canada; Keenan Research Centre of Biomedical Science and Li Ka Shing Knowledge Institute, St. Michael’s Hospital of Unity Health Toronto, 209 Victoria Street, Toronto M5B 1X3, ON, Canada; Division of Cardiac Surgery, St. Michael’s Hospital of Unity Health Toronto, 30 Bond Street, Toronto M5B 1W8, ON, Canada; Keenan Research Centre of Biomedical Science and Li Ka Shing Knowledge Institute, St. Michael’s Hospital of Unity Health Toronto, 209 Victoria Street, Toronto M5B 1X3, ON, Canada; Division of Endocrinology and Metabolism, St. Michael’s Hospital of Unity Health Toronto, Toronto, ON, Canada; Keenan Research Centre of Biomedical Science and Li Ka Shing Knowledge Institute, St. Michael’s Hospital of Unity Health Toronto, 209 Victoria Street, Toronto M5B 1X3, ON, Canada; Department of Pharmacology and Toxicology, University of Toronto, 1 King's College Circle, Toronto M5S 3K3, ON, Canada; Department of Physiology and Pharmacology, Western University, London, ON, Canada; Molecular Medicine Research Laboratories, Robarts Research Institute, London, ON, Canada; Division of Cardiac Surgery, St. Michael’s Hospital of Unity Health Toronto, 30 Bond Street, Toronto M5B 1W8, ON, Canada; Keenan Research Centre of Biomedical Science and Li Ka Shing Knowledge Institute, St. Michael’s Hospital of Unity Health Toronto, 209 Victoria Street, Toronto M5B 1X3, ON, Canada; Department of Pharmacology and Toxicology, University of Toronto, 1 King's College Circle, Toronto M5S 3K3, ON, Canada; Department of Surgery, University of Toronto, 149 College Street, Toronto M5T 1P5, ON, Canada

**Keywords:** Semaglutide, GLP-1 receptor agonists, Progenitor cell, Atherosclerosis, Cardiometabolic disease, Obesity

## Abstract

**Background and Aims:**

Glucagon-like peptide-1 receptor agonists (GLP-1RAs) reduce major atherosclerotic cardiovascular events in individuals living with either diabetes or obesity. Since the turnover of vascular regenerative (VR) stem and progenitor cells has been demonstrated to modulate vessel repair and atherothrombotic risk, this study aimed to determine the effect of the GLP-1RA semaglutide on the levels of circulating VR cells.

**Methods:**

SEMA-VR CardioLink-15 was a randomized translational trial of usual care vs semaglutide for 6 months in 46 participants with either type 2 diabetes and/or obesity plus atherosclerotic cardiovascular disease (ASCVD) or ASCVD risk factors. Vascular regenerative cells were enumerated using multi-parametric flow cytometry for high aldehyde dehydrogenase activity (ALDH^hi^) and lineage-specific cell surface marker expression. The primary endpoint was the 6-month change in VR cell content.

**Results:**

Compared with usual care (*n* = 24), semaglutide (*n* = 22) led to a greater increase in the number of VR cells [high aldehyde dehydrogenase 1A1 activity and low side scatter (ALDH^hi^SSC^low^): +0.8% vs +34.8%; *P* = .036], pan-haematopoietic myeloid progenitors (ALDH^hi^SSC^low^CD45^+^: +2.8% vs +40.1%; *P* = .017), and endothelial precursors (ALDH^hi^SSC^low^CD34^+^ CD133^+^ CD45^−^: −2.3% vs +66.2%; *P* = .037) from baseline. Semaglutide also decreased granulocyte precursors (ALDH^hi^SSC^hi^: +0.3% vs −50.8%; *P* = .002), particularly those expressing the neutrophil activation marker CD66b and chemokine receptor CXCR2. Semaglutide down-regulated serum proteins over-represented in pro-inflammatory tumour necrosis factor and interleukin signalling pathways.

**Conclusions:**

In people living with either type 2 diabetes or obesity plus ASCVD risk, semaglutide increased circulating VR cell content while reducing pro-inflammatory granulocyte precursors and cytokine production. Collectively, these findings suggest that semaglutide may improve endogenous progenitor cell–mediated blood vessel repair processes.

## Structured Graphical Abstract

**Structured Graphical Abstract:**
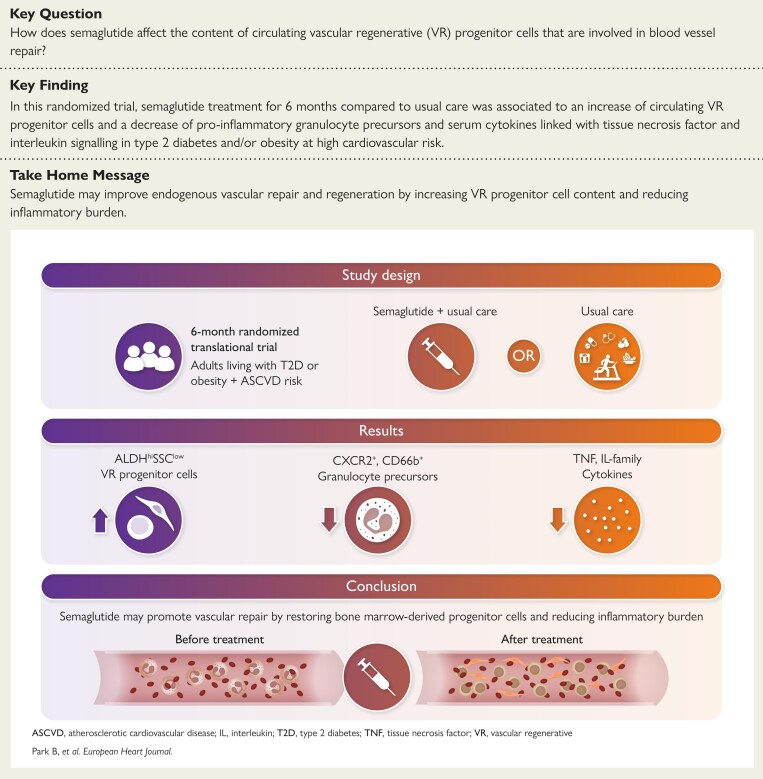
The SEMA-VR CardioLink-15 Translational Study. ALDH^hi^SSC^low^, high aldehyde dehydrogenase 1A1 activity and low side scatter; ASCVD, atherosclerotic cardiovascular disease; IL, interleukin; T2D, Type 2 diabetes; TNF, tumour necrosis factor; VR, vascular regenerative. Generated with a licensed version of BioRender.com.

**See the editorial comment for this article ‘Incretin therapy for cardiovascular disease: opportunities, mechanistic gaps, and challenges’, by D. Raaschou-Oddershede and N. Sattar, https://doi.org/10.1093/eurheartj/ehaf983.**

Translational perspective SEMA-VR CardioLink-15 was a randomized translational trial aimed to determine the effect of semaglutide on bone marrow–derived progenitor cells, as a potential mechanism underlying the cardioprotective properties of semaglutide. The study found that in people living with type 2 diabetes and/or obesity, 6-month semaglutide treatment, compared with usual care, led to a greater increase in the content of circulating vascular regenerative progenitor cells, including myeloid progenitors and endothelial precursors. Semaglutide also decreased granulocyte precursors and serum proteins involved in pro-inflammatory signalling. These findings reveal a previously unrecognized biological action of semaglutide that involves bone marrow progenitor–mediated endogenous vascular repair mechanisms.

## Introduction

Atherosclerotic cardiovascular disease (ASCVD) remains the leading global cause of death, accounting for over 12 million deaths annually.^1^ Individuals with type 2 diabetes (T2D) and obesity face a disproportionately high risk of ASCVD due to overlapping cardiometabolic perturbations. These include chronic low-grade inflammation, oxidative stress, insulin resistance, dyslipidaemia, endothelial dysfunction, pro-thrombotic states, altered adipokine signalling, and maladaptive neurohormonal activation—all of which converge to accelerate vascular injury, plaque progression, and adverse cardiovascular events.^2–7^

Semaglutide is a glucagon-like peptide-1 receptor agonist (GLP-1RA) that is indicated for glycaemic control in T2D and weight reduction in people living with obesity. A meta-analysis of 11 large (*n* ≥ 500) cardiovascular and kidney outcome trials demonstrated a 14% reduction in major adverse cardiovascular events (MACE) with GLP-1RA therapy, including relative risk reductions of 14% for cardiovascular death, 10% for non-fatal myocardial infarction, and 13% for non-fatal stroke.^8^ Subcutaneous semaglutide specifically reduced MACE by 26% in individuals living with T2D^9^ and by 20% in those without diabetes but with ASCVD and a body mass index (BMI) > 27 kg/m^2^.^10^ Cardioprotective effects were consistent across diverse populations, including those with a history of prevalent heart failure^11^ and coronary artery bypass grafting.^12^ More recently, the SOUL trial (*n* = 9650) demonstrated a 14% reduction in MACE with oral semaglutide (14 mg/day) among individuals living with T2D and high cardiovascular risk.^13^ Despite compelling clinical trial evidence, the underlying biological pathways by which semaglutide exerts its cardiovascular benefits remain incompletely elucidated in humans.

Vascular regenerative (VR) progenitor cells are a rare population of circulating bone marrow–derived cells, including myeloid haematopoietic progenitors and endothelial precursors, that are involved in endogenous vascular repair and regeneration.^2,5,14–23^ Earlier studies demonstrated that the flux of circulating progenitor cells are inversely linked to cardiovascular events.^24–28^ We previously found that individuals with ASCVD risk factors, including T2D, obesity, or those of South Asian ethnicity, exhibited depletion of multiple VR progenitor cell subsets relative to age- and sex-matched controls.^16–18^ We have also reported restoration of VR progenitor cell content following pharmacologic treatment with empagliflozin^19,29^ and icosapent ethyl^20^ as well as post-bariatric surgery.^17^ Collectively, our work to date has identified defective vascular reparative bone marrow progenitor cell flux as a central transducer of cardiometabolic risk and a viable pharmacological target, with mechanistic improvements demonstrated in linked clinical trials.

The objective of this study was to evaluate the effect of semaglutide on circulating VR progenitor cell content and serum inflammatory protein expression. We hypothesized that once-weekly subcutaneous semaglutide, compared with usual care, for 6 months would increase VR progenitor cell content in individuals at elevated ASCVD risk and concurrently modulate the levels of inflammatory cytokines.

## Methods

### Trial design

The Semaglutide and Vascular Regeneration Study (SEMA-VR CardioLink-15; NCT05870462) was a 6-month, open-label, randomized translational trial. The protocol was approved by the Advarra Central Institutional Review Board (Columbia, MD, USA), and all participants provided written informed consent prior to trial entry. Eligible participants were aged ≥18 years with either a history of ASCVD or ≥2 risk factors for ASCVD, plus documented T2D, BMI ≥ 30 kg/m^2^, or BMI ≥ 27 kg/m^2^ if the participant presented with ≥1 weight-related comorbidity. Individuals currently using or with prior exposure to GLP-1RAs were excluded.

Participants were randomized 1:1 to receive either usual care or semaglutide. Semaglutide was initiated at 0.25 mg/week with planned dose increments every 4 weeks to a final target dose of 1.0 mg/week over the 6-month period. Randomization was conducted using a computer-generated sequence with block sizes of four to eight. Sample size was calculated using a two-sample *t*-test based on prior estimates of treatment effect on VR cell frequency.^17,19^ A total of 34 participants per arm was determined to provide >80% power to detect a 7.2% difference in mean VR cell content (α = .05).

There were two in-person clinic visits (baseline and 6 months) and two interim telephone follow-ups (at 1 and 2 months). Baseline assessments included medical history, anthropometric measurements, and laboratory values that included haemoglobin A1c (HbA1c), lipid profile, and estimated glomerular filtration rate (eGFR). Peripheral venous blood (20 mL) was collected at baseline and 6 months; 10 mL was processed immediately for flow cytometry and 10 mL for sera that were stored at −80°C and batch processed for proteomic analyses. Participants who experienced adverse effects with semaglutide 1.0 mg/week had the option to have their dose reduced to 0.5 mg/week at the discretion of the study doctor. Individuals who discontinued semaglutide during dose escalation were retained in the analysis if they completed the final study visit.

The primary outcome was the 6-month change in the content of primitive progenitor cells with high aldehyde dehydrogenase 1A1 activity and low side scatter (ALDH^hi^SSC^low^)—a population enriched for early haematopoietic progenitors and endothelial precursors with vessel regenerative potential.^30–32^ Secondary outcomes included changes in the content of ALDH^hi^SSC^mid^ monocytes and ALDH^hi^SSC^hi^ granulocyte precursors. We also measured changes in intracellular oxidative stress within ALDH^hi^ cell subsets and the serum concentrations of 89 proteins that are involved in cytokine signalling and immune surveillance.

### Multi-parametric flow cytometry

Peripheral blood mononuclear cells (PBMCs) were isolated using SepMate^TM^ tubes (STEMCELL Technologies, Vancouver, Canada). Aldehyde dehydrogenase activity—an antioxidant and cytoprotective enzyme highly expressed in progenitor cells of mesodermal lineages^33^—was quantified using the ALDEFLUOR™ kit (STEMCELL Technologies). Three cell subsets were established: ALDH^hi^SSC^low^ primitive progenitors, ALDH^hi^SSC^mid^ monocytes, and ALDH^hi^SSC^hi^ granulocyte precursors (see Supplementary data online, *Figure S1*) and stained with a panel of primitive vs lineage-specific antibodies as previously described (see Supplementary data online, *Table S1*).^34^ Intracellular oxidative stress was assessed using CellROX^TM^ (Invitrogen, Waltham, USA), a cell-permeant fluorogenic probe that detects cytosolic reactive oxygen species (ROS). All samples were analysed on a CytoFLEX LX flow cytometer (Beckman Coulter, Indianapolis, IN, USA).

### Olink® proteomic analysis

Serum concentrations of inflammation- and immunity-related proteins were quantified using the Olink® Target 48 Cytokine and Target 48 Immune Surveillance panels (Olink® Proteomics AB, Uppsala, Sweden). Eighty-nine oligonucleotide-labelled antibody probe pairs were added to 20 μL of serum, along with internal and external controls. Quantification was performed by real-time PCR on the Olink® Signature Q100 platform, and protein concentrations were reported in pg/mL.

### Statistical analysis

Flow cytometry data were analysed using GraphPad Prism (v10.1.1, GraphPad Software, San Diego, CA, USA). Percent change from baseline was calculated as [(6-month value − baseline value)/baseline value] × 100%. Between-group comparisons were made using unpaired two-tailed *t*-tests and analysis of covariance (ANCOVA) to adjust for baseline differences. A modified intention-to-treat analysis was applied: the usual care group comprised participants who did not receive any semaglutide and completed the study, while the semaglutide group included those who received at least one dose of semaglutide and completed the 6-month visit.

Proteomics data were analysed on RStudio Server (v2023.12.0 + 369). Raw absolute concentration values were log_2_-transformed to approximate normal distribution. The *duplicateCorrelation* function from the *limma* package was used to model within-subject time effects and treatment-by-time interactions. Differentially expressed proteins were defined as those with an adjusted *P* < .05 after false discovery rate (FDR) correction. Over-representation analysis was performed using the *clusterProfiler* package (v4.9.0.002), applying a hypergeometric test to identify significantly enriched REACTOME pathways.

## Results

Participants were enrolled between April 2023 and November 2024. A total of 73 individuals were randomized to either usual care (*n* = 37) or semaglutide (*n* = 36) (see Supplementary data online, *Figure S2*). Complete datasets were available for 46 participants: 24 in the usual care group and 22 in the semaglutide group. Of those randomized to semaglutide, 16 reached the full 1.0 mg/week dose, four remained on 0.5 mg/week, and two discontinued semaglutide due to gastrointestinal intolerance. Baseline demographic and clinical characteristics were balanced between the groups and are summarized in *Table 1*.

**Table 1:** Baseline characteristics of SEMA-VR CardioLink-15 trial participants

Characteristic	Usual care (*n* = 24)	Semaglutide (*n* = 22)	*P*
Female, no. (%)	11 (46)	9 (41)	.774
Age, years	54.7 ± 10.9	58.9 ± 8.9	.165
Body weight, kg	75.7 ± 15.2	84.2 ± 17.0	.079
BMI, kg/m^2^	28.6 ± 4.6	30.6 ± 5.2	.165
Systolic BP, mmHg	131.0 ± 19.5	135.6 ± 18.8	.412
Diastolic BP, mmHg	78.5 ± 8.9	79.8 ± 8.8	.616
HbA1c, %	7.8 ± 1.4	7.6 ± 1.2	.457
Total cholesterol	4.2 ± 1.3	4.1 ± 1.7	.768
LDL-C, mmol/L	2.3 ± 1.1	2.1 ± 1.3	.721
HDL-C, mmol/L	1.1 ± .4	1.3 ± .5	.273
TG, mmol/L	1.8 ± 1.4	1.5 ± 0.9	.426
eGFR, mL/min/1.73 m^2^	95.4 ± 13.6	91.1 ± 17.2	.381
eGFR > 60 mL/min/1.73 m^2^, no. (%)	21 (88)	20 (91)	>.999
Type 2 diabetes, no. (%)	21 (88)	19 (86)	>.999
Duration of type 2 diabetes, years	8.1 ± 7.2	11.1 ± 9.7	.186
ASCVD, no. (%)	6 (25)	6 (27)	>.999
Revascularization, no. (%)	4 (17)	8 (36)	.183
Current or past smoker, no. (%)	6 (25)	7 (32)	.746
Medications			
Statin, no. (%)	18 (75)	17 (77)	>.999
Ezetimibe, no. (%)	2 (8)	2 (9)	>.999
ACEi/ARB, no. (%)	8 (33)	15 (68)	.**038**
Ca^2+^ channel blocker, no. (%)	1 (4)	3 (14)	.336
Beta blockers, no. (%)	7 (29)	7 (32)	.316
ASA, no. (%)	4 (17)	9 (50)	.103
NOAC/DAPT, no. (%)	4 (17)	3 (14)	>.999
Diuretics, no. (%)	1 (4)	2 (11)	.600
Metformin, no. (%)	18 (75)	15 (68)	.746
Insulin, no. (%)	0 (0)	3 (14)	.101
SGLT2i, no. (%)	9 (38)	8 (36)	>.999
DPP-4i, no. (%)	9 (38)	7 (32)	.763
Sulfonylurea, no. (%)	6 (25)	4 (18)	.725

Bold values highlight *P* < .05 values.

*P*-values (*P*) for categorical variables were calculated using Fisher’s exact test. *P*-values for continuous variables were calculated using an unpaired *t*-test.

ACEi, angiotensin-converting enzyme inhibitor; ARB, angiotensin II receptor blocker; ASA, acetylsalicylic acid; ASCVD, atherosclerotic cardiovascular disease; BMI, body mass index; BP, blood pressure; DAPT, dual antiplatelet therapy; DPP-4i, dipeptidyl peptidase-4 inhibitor; eGFR, estimated glomerular filtration rate; HbA1c, glycated haemoglobin; HDL-C, high-density lipoprotein cholesterol; LDL-C, low-density lipoprotein cholesterol; NOAC, novel oral anticoagulant; SGLT2i, sodium-glucose co-transporter 2 inhibitor; TG, triglycerides.

At 6 months, there was a mean body weight loss of 0.42 kg in the usual care and 3.41 kg in the semaglutide groups (*P* = .062) (see Supplementary data online, *Figure S3*). Mean HbA1c decreased by 0.62% and 0.67% in the usual care and semaglutide groups, respectively (*P* = .84) (see Supplementary data online, *Figure S3*).

A total of six adverse events were reported: one in the usual care group and five in the semaglutide group. The individual in the usual care arm underwent coronary artery bypass graft surgery. Among the semaglutide-assigned participants, two reported gastrointestinal disturbances (both treatment-related), one underwent coronary artery bypass graft surgery, one experienced blurred vision and lower extremity oedema, and one was hospitalized for gallstones and endocarditis.

### Semaglutide increases circulating ALDH^hi^SSC^low^ VR progenitor cells

Mean absolute number and percent changes in VR progenitor cell subsets are reported in *Tables 2* and Supplementary data online, *Table S2*. ALDH^hi^SSC^low^ cells are a well-characterized population of early myeloid haematopoietic progenitors and endothelial precursors with clonogenic potential.^30^ These cells exhibit a potently pro-angiogenic transcriptomic and secretory profile,^31^ home to areas of ischaemia,^30^ and restore perfusion in mice with critical limb ischaemia.^31,32^

**Table 2:** Semaglutide treatment increased the absolute number of circulating ALDH^hi^SSC^low^ vascular regenerative cells and reduced pro-inflammatory ALDH^hi^SSC^hi^ granulocyte precursor cells

	Usual care (*n* = 24)			Semaglutide (*n* = 22)			ANCOVA
	Baseline	6-month	*P*	Baseline	6-month	*P*	
ALDH^hi^SSC^low^	382 ± 59	327 ± 43	.381	276 ± 34	362 ± 50	.**013**	.205
CD34^+^	334 ± 56	297 ± 41	.553	226 ± 32	262 ± 46	.347	.949
CD133^+^	190 ± 39	169 ± 25	.694	172 ± 23	218 ± 43	.250	.315
CD45^+^	305 ± 52	256 ± 32	.343	222 ± 25	300 ± 40	.**017**	.144
CD34^+^ CD133^+^	171 ± 38	155 ± 24	.785	140 ± 21	155 ± 38	.755	.957
CD34^+^ CD133^+^ CD45^−^	91 ± 15	61 ± 13	.092	74 ± 15	97 ± 24	.281	.078
ALDH^hi^SSC^mid^	19 457 ± 2,462	19 123 ± 2,859	.569	21 503 ± 2,564	18 753 ± 2783	.425	.563
CD14^+^	16 250 ± 2228	16 483 ± 2518	.826	17 204 ± 2135	14 858 ± 2054	.397	.370
CD14^+^ CCR2^+^	15 860 ± 2163	16 127 ± 2480	.832	16 787 ± 2090	14 539 ± 2018	.405	.375
CD14^+^ CD86^+^	16 095 ± 2187	16 106 ± 2503	.738	17 043 ± 2130	14 700 ± 2038	.394	.404
CD14^+^ CD163^+^	12 962 ± 1814	13 723 ± 2199	.980	14 133 ± 1985	11 588 ± 1714	.332	.253
CD14^+^ CD36^+^	15 970 ± 2195	15 897 ± 2302	.688	16 997 ± 2146	14 715 ± 2043	.408	.427
ALDH^hi^SSC^hi^	8 379 ± 3244	6 016 ± 1596	.315	12 810 ± 2064	6620 ± 1842	**<**.**001**	.293
CD49d^+^	2832 ± 416	3070 ± 593	.636	5270 ± 937	3598 ± 1082	.**014**	.070
CD66b^+^	7546 ± 3233	5364 ± 1554	.361	11 901 ± 1967	6044 ± 1789	**<**.**001**	.332
CXCR2^+^	429 ± 123	373 ± 105	.504	917 ± 275	189 ± 66	.**015**	.071

Bold values highlight *P* < .05 values.

Data are presented as mean absolute count of cells per 10 mL peripheral blood sample ± SEM.

Paired *t*-tests were performed to evaluate the differences in absolute counts from baseline to 6 months within each group. Analysis of covariance was performed to assess the 6-month effect of semaglutide on absolute counts after adjusting for baseline differences.

ALDH, aldehyde dehydrogenase; SSC, side scatter.

**Figure 1:**
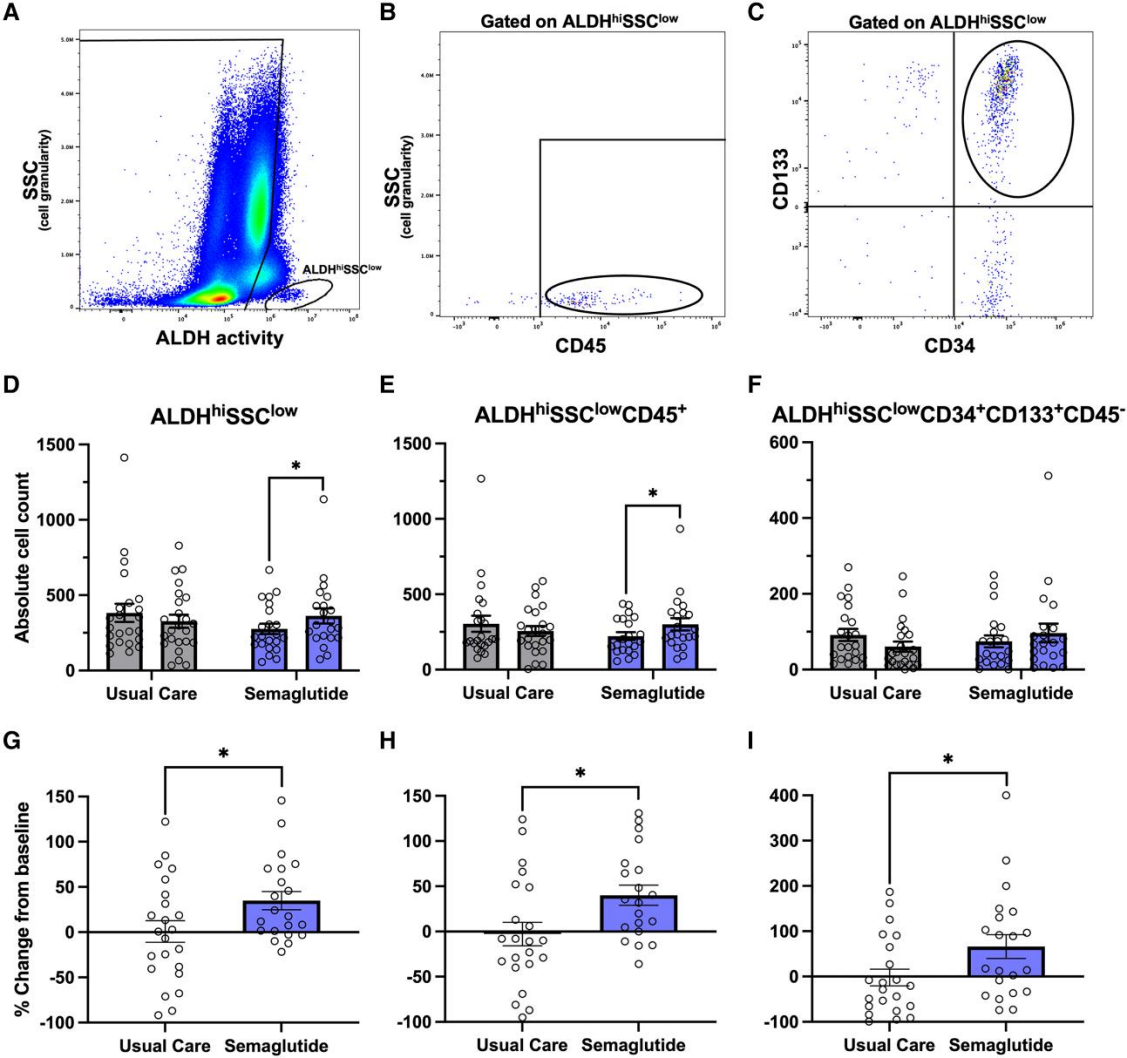
Semaglutide increases circulating ALDH^hi^SSC^low^ VR progenitor cells of myeloid and endothelial lineages. Representative flow cytometry plots illustrating gating strategies for (*A*) ALDH^hi^SSC^low^ VR progenitor cells, (*B*) ALDH^hi^SSC^low^ CD45^+^ myeloid progenitors, and (*C*) ALDH^hi^SSC^low^ CD34^+^ CD133^+^ CD45^−^ putative endothelial precursors. Absolute counts of each cell population at baseline and 6 months following assignment to usual care (*n* = 24) or subcutaneous semaglutide (*n* = 22). Semaglutide was associated with a significant increase in (*D*) ALDH^hi^SSC^low^ and (*E*) CD45+ subsets and a trend towards increase in the (*F*) CD34^+^ CD133^+^ CD45^−^ endothelial precursor population. Between-group comparisons using analysis of covariance, adjusted for baseline values, did not reach statistical significance. Percent change from baseline to 6 months in circulating (*G*) ALDH^hi^SSC^low^ progenitor cells, (*H*) CD45^+^ myeloid progenitors, and (*I*) CD34^+^ CD133^+^ CD45^−^ endothelial precursors. Percent increases were significantly greater with semaglutide vs usual care in *G–I*. *P < .05. Data are presented as mean ± SEM. ALDH, aldehyde dehydrogenase; ANCOVA, analysis of covariance; SSC, side scatter; VR, vascular regenerative

The absolute number of circulating ALDH^hi^SSC^low^ cells increased significantly after 6 months of semaglutide treatment (*P* = .013) (*Table 2*; *Figure 1D*). This corresponded to a greater percent increase in ALDH^hi^SSC^low^ cells in the semaglutide vs usual care groups (+34.8% vs +0.8%; *P* = .036) (see Supplementary data online, *Table S2*; *Figure 1G*). Numerical increases were also observed in the ALDH^hi^SSC^low^ cell subsets expressing the pan-haematopoietic marker CD45 and in putative endothelial precursor cells (ALDH^hi^SSC^low^CD34^+^ CD133^+^ CD45^−^) (*P* < .05 for both comparisons) (*Table 2*; *Figure 1E, F, H*, and *I*). There was no significant between-group difference in the subset co-expressing CD34 and CD133 (*Table 2*; Supplementary data online, *Table S2*). These findings suggest that semaglutide may enhance the circulating pool of pro-angiogenic myeloid haematopoietic progenitor cells (HPCs) and putative endothelial precursor cells (EPCs) with vessel-integrating capacity.^35^

### Semaglutide does not modulate ALDH^hi^SSC^mid^ monocyte content or polarization

Monocytic lineage progenitors were identified based on high ALDH activity and intermediate side scatter properties (ALDH^hi^SSC^mid^), alongside expression of the pan-monocyte marker CD14.^36,37^ Further phenotyping was performed using antibodies directed against CD163, CCR2, CD36, and CD86, which respectively denote anti-inflammatory,^38^ migratory;^39,40^ lipid-scavenging;^41^ and pro-inflammatory functional subsets.^42^

Semaglutide treatment did not modulate the absolute number of ALDH^hi^SSC^mid^ monocytes relative to usual care (*Table 2*). Similarly, the content of monocyte subsets stratified by CD163, CCR2, CD36, or CD86 expression remained unchanged. Adjustment for baseline variation using ANCOVA did not yield statistically significant between-group differences in circulating monocyte populations. These findings suggest that semaglutide may not actively modulate the content or phenotypic polarization of ALDH^hi^SSC^mid^ monocyte progenitors.

### Semaglutide reduces circulating ALDH^hi^SSC^hi^ pro-inflammatory granulocyte precursor burden

Granulocyte precursors were defined as ALDH^hi^SSC^hi^ cells and further characterized by co-expression of CXCR2, CD66b, and CD49d. CXCR2 is a chemokine receptor involved in recruitment of neutrophils and macrophages to the endothelial wall during atherosclerosis.^43–45^ CD66b is involved in neutrophil activation and is commonly detected within atherosclerotic plaques.^45^ Granulocytes expressing the integrin receptor CD49d were previously identified to be capable of augmenting endothelial adherence and vessel repair.^46^

**Figure 2:**
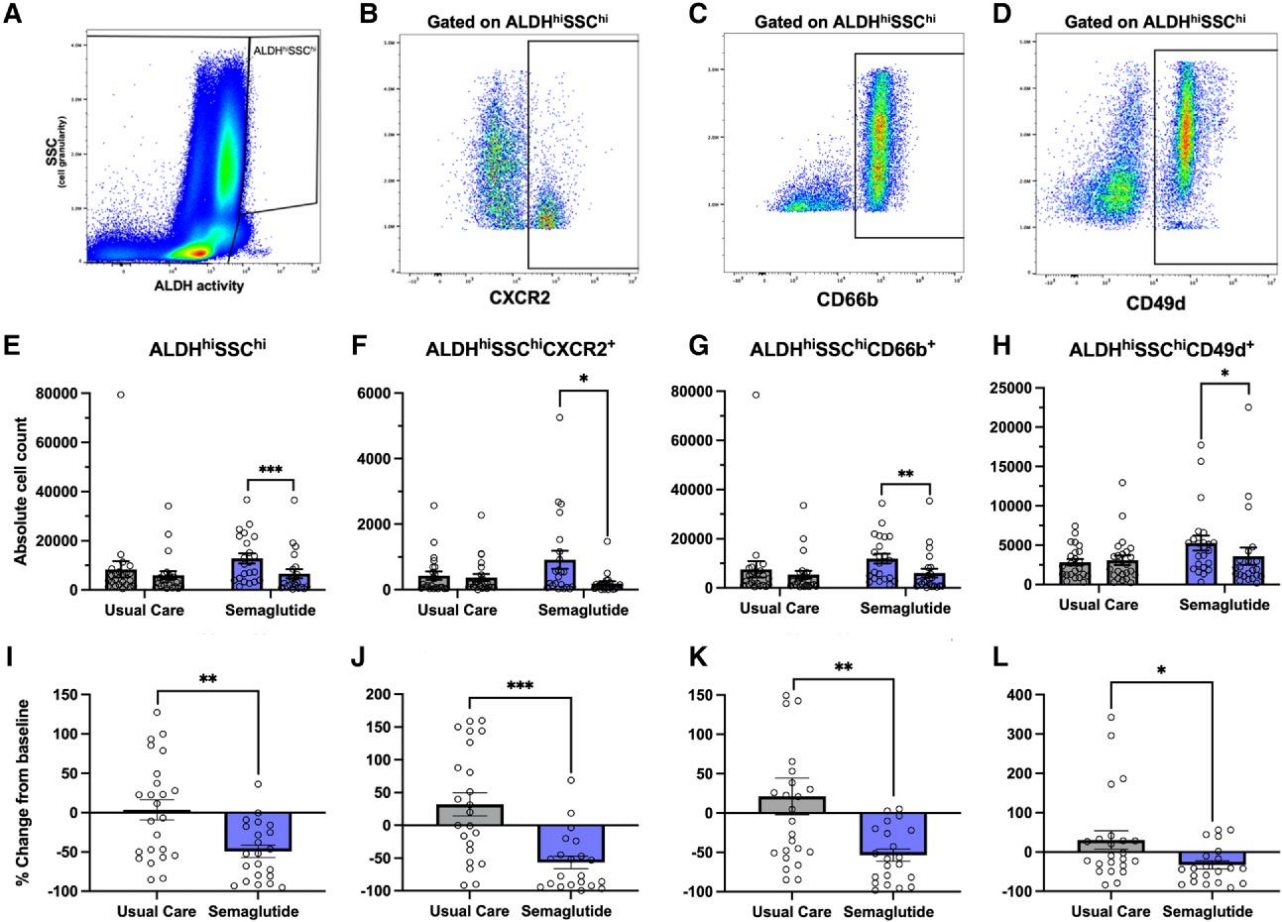
Semaglutide reduces circulating ALDH^hi^SSC^hi^ granulocyte precursors and lineage-specific pro-inflammatory subsets. Representative flow cytometry plots illustrating gating strategies for (*A*) ALDH^hi^SSC^hi^ granulocyte precursors and subpopulations expressing (*B*) CXCR2 (chemotactic receptor), (*C*) CD66b (neutrophil activation marker), and *D*) CD49d (adhesion molecule associated with vascular repair). Absolute counts of each granulocyte subset at baseline and 6 months following assignment to usual care (*n* = 24) or subcutaneous semaglutide (*n* = 22). Significant reductions in (*E*) ALDH^hi^SSC^hi^ granulocyte precursors, as well as (*F*) CXCR2^+^, (*G*) CD66b^+^, and (*H*) CD49d^+^ subsets, were observed after semaglutide treatment. Between-group comparisons using analysis of covariance, adjusted for baseline values, did not reach statistical significance. Percent change from baseline in (*I*) ALDH^hi^SSC^hi^ granulocyte precursors, as well as (*J*) CXCR2^+^, (*K*) CD66b^+^, and (*L*) CD49d^+^ subsets. All ALDH^hi^SSC^hi^ cell subsets demonstrated significantly greater reductions with semaglutide compared with usual care. *P < .05; **P < .01; ***P < .001. ALDH, aldehyde dehydrogenase; ANCOVA, analysis of covariance; CXCR2, CXC chemokine receptor 2; SSC, side scatter; VR, vascular regenerative

Individuals treated with semaglutide exhibited a significant two-fold reduction in the absolute count of ALDH^hi^SSC^hi^ granulocyte precursors at 6 months vs baseline (*P* < .001) (*Table 2*; *Figure 2E*). This decline corresponded to a greater percent decrease in ALDH^hi^SSC^hi^ granulocyte precursors with semaglutide compared with usual care (−50.8% semaglutide vs +0.3% usual care; *P* = .002) (see Supplementary data online, *Table S2*; *Figure 2I*). Subpopulations co-expressing CXCR2, CD66b, and CD49d similarly declined within the semaglutide group at 6 months (*P* < .05) (*Table 2*; *Figure 2F–H*), with all showing a significantly greater reduction compared with usual care (*P* < .05) (see Supplementary data online, *Table S2*; *Figure 2J–L*). However, ANCOVA analyses did not yield any statistically significant between-group differences after adjusting for baseline values (*Table 2*; *Figure 2E–H*). Together, these data suggest that semaglutide may attenuate circulating granulocyte activity, potentially through the suppression of activated and migratory neutrophil precursors.

### Semaglutide did not alter intracellular oxidative stress content within VR cell subsets

**Figure 3:**
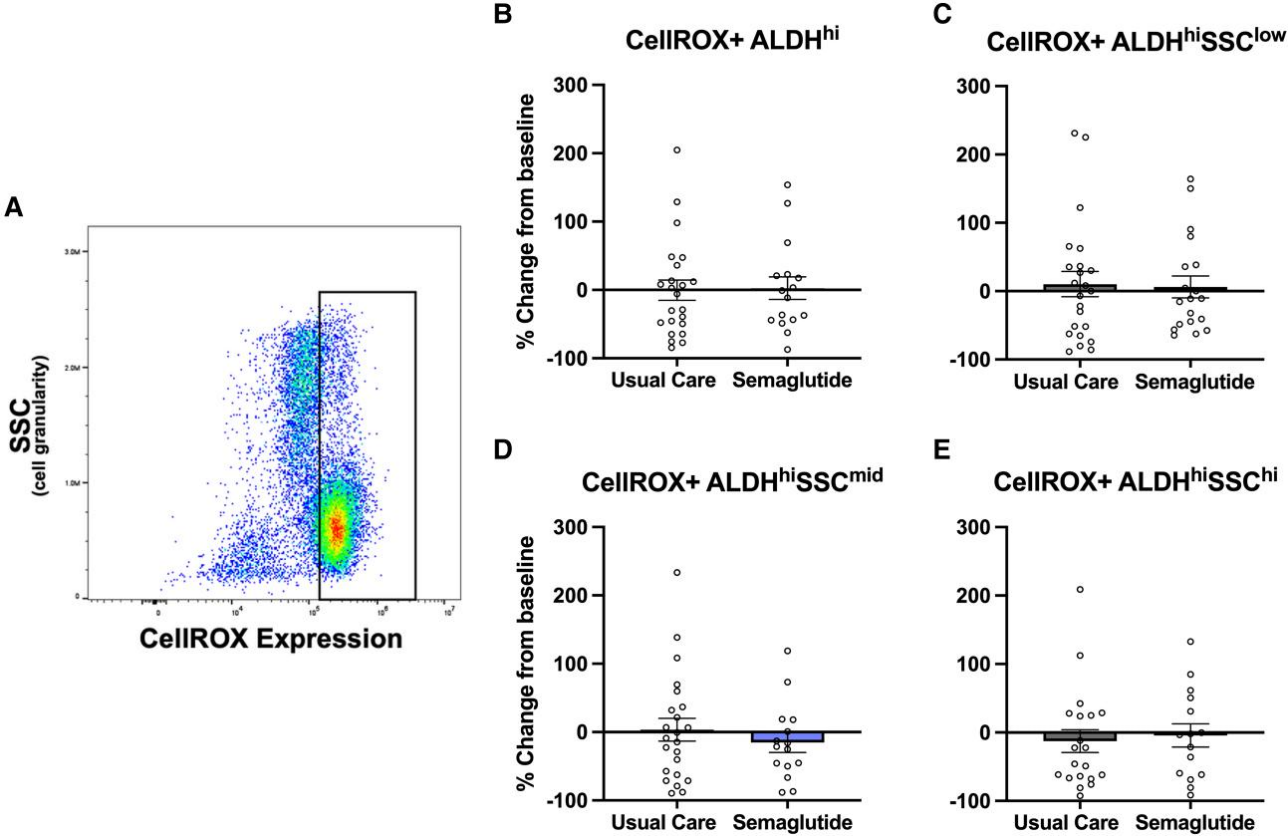
Semaglutide treatment does not alter intracellular reactive oxygen species content in ALDH^hi^ cell subsets. (*A*) Representative flow cytometry plot showing detection of intracellular reactive oxygen species using CellROX™ Red. (*B–E*) Percent change from baseline (mean ± SEM) in reactive oxygen species content 6 months following assignment to usual care (*n* = 24) or subcutaneous semaglutide (*n* = 22). No significant differences in (*B*) total ALDH^hi^ cells, (*C*) ALDH^hi^SSC^low^ VR progenitor cells, (*D*) ALDH^hi^SSC^mid^ monocytes, and (*E*) ALDH^hi^SSC^hi^ granulocyte precursors were observed between the treatment arms. These data suggest that semaglutide does not significantly modulate oxidative stress within circulating progenitor and precursor cell subsets. ALDH, aldehyde dehydrogenase; ROS, reactive oxygen species; SSC, side scatter

Reactive oxygen species content in ALDH^hi^SSC^low^ VR progenitor cells, ALDH^hi^SSC^mid^ monocytes, and ALDH^hi^SSC^hi^ granulocyte precursors remained unchanged following semaglutide treatment when compared with usual care (*Figure 3B–E*). A representative flow cytometric ROS plot in bulk ALDH^hi^ cells is shown in *Figure 3A*. These findings suggest that 6-month semaglutide therapy did not significantly impact intracellular oxidative stress within ALDH^hi^ cell subsets.

### Semaglutide modulates serum protein expression linked to cytokine and immune signalling

Proteomic profiling identified 14 proteins with significantly altered expression between baseline and 6 months in the semaglutide group (*Table 3*). Most of these markers are associated with either angiogenesis (VEGFR2, FGF21) or inflammation (CD80, IFNL1, IL1RN, IL17C, IL17D, IL32, LIF, TNFRSF4, TNFRSF9, TNFSF14). Between-group comparisons did not yield statistically significant differences after multiple-testing correction (adjusted *P* > .05), suggesting that while semaglutide influences circulating cytokine profiles, these effects may be modest in magnitude or masked by inter-individual variability.

**Table 3:** Differentially expressed serum proteins following semaglutide treatment identified by targeted proteomic profiling

Protein	log_2_FC	*P*	Cellular action
VEGFR2	−.2770	.0001	Receptor for VEGF-A, VEGF-C, and VEGF-D. Regulates angiogenesis, vascular development, vascular permeability, and embryonic haematopoiesis
TNFRSF4	−.3040	.0060	Activates NF-kB and suppresses apoptosis by promoting expression of apoptosis inhibitors BCL2 and BCL2L1/BCL2-XL
KRT18	−.8038	.0083	Type I intermediate filament chain involved in uptake of thrombin–antithrombin complexes by hepatic cells
IFNL1	−.7162	.0083	Involved in antiviral host defence in epithelial tissues. Induces expression of IFN-stimulated genes by JAK/STAT activation
LIF	−.4230	.0099	Polyfunctional cytokine involved in cell survival, proliferation, and differentiation
FASLG	−.2584	.0114	Involved in immune system regulation, including T-cell development and cytotoxic T-cell or natural killer cell–mediated apoptosis
IL17C	.5725	.0192	Involved in innate immunity of the epithelium. Stimulates TNF-a and IL-1B release through NF-kB and MAPK pathway activation
IL1RN	−.3533	.0192	Anti-inflammatory antagonist of IL-1 family of pro-inflammatory cytokines. Protects from immune dysregulation and uncontrolled systemic inflammation
CD80	−.2858	.0199	Receptor for CD28. Induces T-cell proliferation and cytokine production via NF-kB and MAPK pathway stimulation
IL32	−.3406	.0199	Induces cytokines such as TNF-a and IL8, particularly in macrophages, through activation of NF-kB and MAPK pathways
IL17D	−.2344	.0206	Induces endothelial cell production of IL6, IL8, and GM-CSF
TNFRSF9	−.2588	.0213	Enhances T-cell survival and mitochondrial activity
TNFSF14	−.3151	.0284	Ligand for TNFRSF14. Delivers costimulatory signals to T cells, leading to T-cell proliferation and IFNG production
FGF21	−.7117	.0406	Secreted endocrine factor that stimulates glucose uptake in adipocytes via induction of GLUT1 expression

Serum samples were analysed using the Olink® Target 48 Cytokine and Target 48 Immune Surveillance panels. Proteins with ≤ 30% missing data and significantly altered expression at 6 months compared with baseline (adjusted *P* < .05 after correction for multiple comparisons) are reported. Quantification was performed using the Olink NPX (Normalized Protein Expression) system and analysed in RStudio Server.

BCL, B-cell lymphoma 2; CXCL12, C-X-C motif chemokine 12, also known as stromal cell-derived factor 1 (SDF-1); FASLG, Fas ligand; FGF2, fibroblast growth factor 2; FGF21, fibroblast growth factor 21; FLT1, Fms-like tyrosine kinase 1, also known as vascular endothelial growth factor receptor 1 (VEGFR1); GLUT1, glucose transporter protein type 1; GM-CSF, granulocyte-macrophage colony-stimulating factor; HAVCR1, hepatitis A virus cellular receptor 1, also known as T-cell immunoglobulin and mucin domain 1 (TIM-1); IFNG, interferon gamma; IFNL1, interferon lambda 1, also known as interleukin 29 (IL-29); IL17C, interleukin 17-C; IL17D, interleukin 17-D; IL1RN, interleukin-1 receptor antagonist; IL32, interleukin 32; IL4R, interleukin-4 receptor alpha chain; JAK, Janus kinase; KRT18, keratin 18; LIF, leukaemia inhibitory factor; NF-kB, nuclear factor kappa-light-chain-enhancer of activated B cells; PGF, placental growth factor; STAT, signal transducers and activators of transcription; TNFSF14, tumour necrosis factor superfamily member 14; TNFRSF4, tumour necrosis factor receptor superfamily member 4, also known as CD134; TNFRSF9, tumour necrosis factor receptor superfamily member 9, also known as CD137 and 4-1BB; TNFRSF14, tumour necrosis factor receptor superfamily member 14; VEGF, vascular endothelial growth factor; VEGFR2, vascular endothelial growth factor receptor 2.

**Figure 4:**
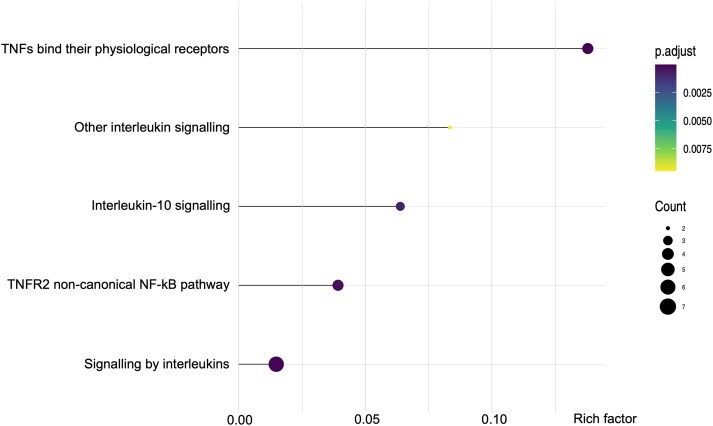
Semaglutide treatment enriches tumour necrosis factor and interleukin signalling pathways at 6 months in the SEMA-VR CardioLink-15 trial. Paired serum samples collected at baseline and 6-month follow-up from participants receiving subcutaneous semaglutide (*n* = 22) were analysed by proximity extension assay targeting 89 proteins across the Olink® Target 48 Cytokine and Target 48 Immune Surveillance panels. Fourteen differentially expressed proteins were identified and subjected to over-representation analysis using the clusterProfiler R package with REACTOME as the annotation source. Five biological pathways were significantly enriched at follow-up compared with baseline (adjusted *P* < .05; *q* < .20), indicating semaglutide-induced modulation of inflammatory and tumour necrosis factor-related signalling networks. NF-kB, nuclear factor kappa-light-chain-enhancer of activated B cells; TNF, tumour necrosis factor; TNFR2, tumour necrosis factor receptor 2

Over-representation analysis (ORA) of the 14 semaglutide-responsive proteins identified five enriched pathways: ‘TNFs bind their physiological receptors’, ‘Signalling by interleukins’, ‘TNFR2 non-canonical NF-κB pathway’, ‘Interleukin-10 signalling’, and ‘Other interleukin signalling (*Figure 4*). These findings suggest that semaglutide may exert immunomodulatory effects by influencing key inflammatory signalling cascades, particularly those governed by tumour necrosis factor (TNF) and interleukin family members.

## Discussion

The SEMA-VR CardioLink-15 translational trial has revealed a previously unrecognized biological action of GLP-1 receptor agonism—specifically, that semaglutide may modulate vascular repair mechanisms through altering the flux of bone marrow–derived progenitor cells. While semaglutide did not affect the content of ALDH^hi^SSC^mid^ monocytic progenitors, it was associated with a reduction in circulating ALDH^hi^SSC^hi^ granulocyte precursors, including migratory and activated neutrophil precursor subsets, alongside differential regulation of serum proteins linked to TNF and interleukin signalling pathways (*Structured Graphical Abstract*). This coordinated shift could suggest a potential rebalancing of the reparative cell milieu towards a less inflammatory, pro-regenerative state. The concept that pharmacological therapy can enhance vascular health not only by lowering traditional cardiometabolic risk factors but also by modulating progenitor cell dynamics and systemic immune signalling represents a novel mechanistic paradigm—one that broadens our understanding of GLP-1RA-mediated cardiovascular protection and identifies bone marrow progenitor flux as a plausible therapeutic target in cardiometabolic disease.

Semaglutide increased the ALDH^hi^SSC^low^ progenitor population, including both haematopoietic (CD45^+^) and endothelial (CD45^−^) lineages. ALDH^hi^SSC^low^ cells have been identified in preclinical models as multipotent progenitors capable of myeloid and lymphoid reconstitution,^47^ secrete pro-angiogenic cytokines, home to ischaemic vascular beds,^48^ and possess a rare subset of putative EPCs that proliferate under endothelial colony-forming cell (ECFC) culture conditions.^30,32^ The expansion of this subset in response to semaglutide highlights the possibility that semaglutide may be involved in mediating vascular repair and angiogenesis.

Our findings align with prior data demonstrating GLP-1RA-induced enhancement of circulating progenitor cell content. In people living with T2D, the GLP-1RA dulaglutide increased CD34^+^ CD133^+^ VEGFR2^+^ EPC numbers and improved their proliferation, migration, adhesion, and tubule formation capacities.^49^ A retrospective analysis by our group further demonstrated that individuals on GLP-1RA therapy had higher levels of ALDH^hi^SSC^low^ cells compared with individuals on sodium-glucose co-transporter 2 inhibitor therapy or neither therapy.^21^

The findings herein offer novel mechanistic insight into the recently reported STRIDE trial, wherein semaglutide improved functional capacity and quality of life in patients living with peripheral artery disease (PAD).^50^ A further association between VR cells and the therapeutic effects of semaglutide in PAD is provided by preclinical models of mice with femoral artery ligation–induced hindlimb ischaemia.^23^ In these studies, transplantation of ALDH^hi^SSC^low^ cells increased both local proliferation of endothelial cells and capillary density as well as improved limb usage as measured by paw print intensity, indicating accelerated revascularization and reperfusion of the injured limb.^30,31^ The observed increase in VR cell content with semaglutide may thus contribute to improved microvascular recovery and symptom relief in PAD.

Semaglutide also reduced pro-inflammatory granulocyte precursors identified as ALDH^hi^SSC^hi^ cells and subsets that serve as key mediators of neutrophil activation and recruitment during atherosclerosis. Notably, our proteomics analysis identified 14 semaglutide-responsive serum proteins that were enriched in TNF and interleukin signalling pathways. Wong *et al.* recently suggested a novel GLP-1-mediated gut–brain axis for the suppression of peripheral inflammation, wherein the activation of GLP-1 receptors in the central nervous system, but not haematopoietic or endothelial GLP-1 receptors, led to reductions in plasma TNF-a signalling and systemic inflammation.^51^ This concept aligns well with the systemic anti-inflammatory signature observed in our study, despite unaltered intracellular ROS levels in VR cells.

That ROS content among ALDH^hi^ subsets was unaffected by semaglutide exposure suggests that VR cell expansion is unlikely to be mediated through oxidative stress modulation. The cytoprotective actions of semaglutide in this setting may be mediated via direct GLP-1 receptor interaction or secondary to systemic improvements in glycaemia and inflammation. While the GLP-1 receptor may be expressed on human cord blood–derived HPCs, relative expression compared with other tissues in the body remains inconclusive.^52,53^ A thorough investigation of VR cell expression of GLP-1 receptors and the specific downstream signalling pathways pertaining to cell survival will help delineate the mechanisms of VR cell recovery.

Finally, the increase in VR cell content observed in our trial occurred with minimal placebo-adjusted weight change (−2.99 kg). This contrasts with the more pronounced weight reduction seen in higher-dose semaglutide trials with longer observation periods^54–56^ and supports the hypothesis that VR cell recovery may be partly independent of weight loss.^22^ We previously showed that bariatric surgery, which enhances endogenous GLP-1 secretion,^57^ restored VR cell levels within 3 months post-surgery and prior to significant weight loss in people living with severe obesity (BMI ≥ 40 kg/m^2^).^17^ These data support a GLP-1-dependent, largely weight-independent pathway for progenitor cell recovery.

Our findings should be cautiously interpreted in the context of several limitations. First, the study sample size was modest and did not reach the initially planned enrolment target, in part due to the open-label design, compliance, and logistical challenges around semaglutide access at the time of study conduct. Second, not all participants in the semaglutide arm reached the maximum dose of 1.0 mg/week due primarily to gastrointestinal disturbances. Third, eligibility was restricted to participants with high ASCVD risk who were not previously on a GLP-1RA, to capture a controlled timeframe for VR cell deficiency and recovery. This may limit the generalizability of our findings towards a broader cardiovascular patient population. Fourth, this study was not designed and therefore not powered to collect clinical outcomes. Accordingly, we do not have direct evidence that changes in VR cell flux translate into cardiovascular event reduction. Nonetheless, the results described herein do support the contention for further exploring how changes in VR cell content may impact on clinical outcomes.

In summary, this randomized translational trial uncovers a previously unrecognized role for semaglutide in modulating vascular repair by reshaping bone marrow–derived progenitor cell flux toward an anti-inflammatory, pro-regenerative profile—establishing progenitor cell dynamics as a novel, mechanistically plausible target for cardiometabolic risk reduction. These mechanistic data may provide translational insights into the cardiovascular benefits of semaglutide observed in SUSTAIN-6, PIONEER-6, SELECT, SOUL, and STRIDE.^9,10,13,50,58^

## Supplementary Material

ehaf690_Supplementary_Data

## Declarations

### Data Availability

Data will be made available from the corresponding author upon reasonable request.

### Pre-registered Clinical Trial Number

The pre-registered clinical trial number is NCT05870462.

## References

[1] Mensah GA, Fuster V, Murray CJL, Roth GA. Global burden of cardiovascular diseases and risks collaborators. Global burden of cardiovascular diseases and risks, 1990–2022. J Am Coll Cardiol 2023;82:2350–473. 10.1016/j.jacc.2023.11.007 38092509 PMC7615984

[2] Park B, Bakbak E, Teoh H, Krishnaraj A, Dennis F, Quan A, et al GLP-1 receptor agonists and atherosclerosis protection: the vascular endothelium takes center stage. Am J Physiol Heart Circ Physiol 2024;326:H1159–76. 10.1152/ajpheart.00574.2023 38426865

[3] Romeo S, Vidal-Puig A, Husain M, Ahima R, Arca M, Bhatt DL, et al Clinical staging to guide management of metabolic disorders and their sequelae: a European Atherosclerosis Society consensus statement. Eur Heart J 2025;**46**:3685–3713. 10.1093/eurheartj/ehaf314 PMC12500331 40331343

[4] Pandey A, Khan MS, Patel KV, Bhatt DL, Verma S. Predicting and preventing heart failure in type 2 diabetes. Lancet Diabetes Endocrinol 2023;11:607–24. 10.1016/S2213-8587(23)00128-6 37385290

[5] Terenzi DC, Bakbak E, Teoh H, Krishnaraj A, Puar P, Rotstein OD, et al Restoration of blood vessel regeneration in the era of combination SGLT2i and GLP-1RA therapy for diabetes and obesity. Cardiovasc Res 2023;119:2858–74. 10.1093/cvr/cvae016 38367275

[6] Cosentino F, Verma S, Ambery P, Treppendahl MB, van Eickels M, Anker SD, et al Cardiometabolic risk management: insights from a European Society of Cardiology Cardiovascular round table. Eur Heart J 2023;44:4141–56. 10.1093/eurheartj/ehad445 37448181

[7] Patel KV, Khan MS, Segar MW, Bahnson JL, Garcia KR, Clark JM, et al Optimal cardiometabolic health and risk of heart failure in type 2 diabetes: an analysis from the Look AHEAD trial. Eur J Heart Fail 2022;24:2037–47. 10.1002/ejhf.2723 36280384

[8] Badve SV, Bilal A, Lee MMY, Sattar N, Gerstein HC, Ruff CT, et al Effects of GLP-1 receptor agonists on kidney and cardiovascular disease outcomes: a meta-analysis of randomised controlled trials. Lancet Diabetes Endocrinol 2025;13:15–28. 10.1016/S2213-8587(24)00271-7 39608381

[9] Marso SP, Bain SC, Consoli A, Eliaschewitz FG, Jódar E, Leiter LA, et al Semaglutide and cardiovascular outcomes in patients with type 2 diabetes. N Engl J Med 2016;375:1834–44. 10.1056/NEJMoa1607141 27633186

[10] Lincoff AM, Brown-Frandsen K, Colhoun HM, Deanfield J, Emerson SS, Esbjerg S, et al Semaglutide and cardiovascular outcomes in obesity without diabetes. N Engl J Med 2023;389:2221–32. 10.1056/NEJMoa2307563 37952131

[11] Deanfield J, Verma S, Scirica BM, Kahn SE, Emerson SS, Ryan D, et al Semaglutide and cardiovascular outcomes in patients with obesity and prevalent heart failure: a prespecified analysis of the SELECT trial. Lancet 2024;404:773–86. 10.1016/S0140-6736(24)01498-3 39181597

[12] Verma S, Emerson S, Plutzky J, Kahn SE, Stensen S, Weeke PE, et al Semaglutide improves cardiovascular outcomes in patients with history of coronary artery bypass graft and obesity. J Am Coll Cardiol 2025;85:541–5. 10.1016/j.jacc.2024.11.008 39566870

[13] McGuire DK, Marx N, Mulvagh SL, Deanfield JE, Inzucchi SE, Pop-Busui R, et al Oral semaglutide and cardiovascular outcomes in high-risk type 2 diabetes. N Engl J Med 2025;392:2001–12. 10.1056/NEJMoa2501006 40162642

[14] Bakbak E, Krishnaraj A, Park B, Verma S, Hess DA. Vascular regenerative cells in cardiometabolic disease. Curr Opin Cardiol 2023;38:546–51. 10.1097/HCO.0000000000001089 37668181

[15] Hess DA, Verma S, Bhatt D, Bakbak E, Terenzi DC, Puar P, et al Vascular repair and regeneration in cardiometabolic diseases. Eur Heart J 2022;43:450–9. 10.1093/eurheartj/ehab758 34849704

[16] Terenzi DC, Al-Omran M, Quan A, Teoh H, Verma S, Hess DA. Circulating pro-vascular progenitor cell depletion during type 2 diabetes: translational insights into the prevention of ischemic complications in diabetes. JACC Basic Transl Sci 2018;4:98–112. 10.1016/j.jacbts.2018.10.005 30847424 PMC6390504

[17] Hess DA, Trac JZ, Glazer SA, Terenzi DC, Quan A, Teoh H, et al Vascular risk reduction in obesity through reduced granulocyte burden and improved angiogenic monocyte content following bariatric surgery. Cell Rep Med 2020;1:100018. 10.1016/j.xcrm.2020.100018 33205058 PMC7659601

[18] Krishnaraj A, Bakbak E, Teoh H, Pan Y, Firoz IN, Pandey AK, et al Vascular regenerative cell deficiencies in South Asian adults. J Am Coll Cardiol 2024;83:755–69. 10.1016/j.jacc.2023.12.012 38355246

[19] Hess DA, Terenzi DC, Trac JZ, Quan A, Mason T, Al-Omran M, et al SGLT2 inhibition with empagliflozin increases circulating provascular progenitor cells in people with type 2 diabetes mellitus. Cell Metab 2019;30:609–13. 10.1016/j.cmet.2019.08.015 31477497

[20] Bakbak E, Krishnaraj A, Bhatt DL, Quan A, Park B, Bakbak AI, et al Icosapent ethyl modulates circulating vascular regenerative cell content: the IPE-PREVENTION CardioLink-14 trial. Med 2024;5:1–17. 10.1016/j.medj.2024.03.009 38552629

[21] Park B, Krishnaraj A, Teoh H, Bakbak E, Dennis F, Quan A, et al GLP-1RA therapy increases circulating vascular regenerative cell content in people living with type 2 diabetes. Am J Physiol Heart Circ Physiol 2024;327:H370–6. 10.1152/ajpheart.00257.2024 38874618

[22] Bakbak E, Terenzi DC, Trac JZ, Teoh H, Quan A, Glazer SA, et al Lessons from bariatric surgery: can increased GLP-1 enhance vascular repair during cardiometabolic-based chronic disease? Rev Endocr Metab Disord 2021;22:1171–88. 10.1007/s11154-021-09669-7 34228302

[23] Verma S, Hess DA. GLP-1RAs for peripheral artery disease: a remarkable STRIDE in the right direction. Cell Metab 2025;37:1257–9. 10.1016/j.cmet.2025.05.001 40466625

[24] Muggeridge D, Dodd J, Ross MD. CD34+ progenitors are predictive of mortality and are associated with physical activity in cardiovascular disease patients. Atherosclerosis 2021;333:108–15. 10.1016/j.atherosclerosis.2021.07.004 34340831

[25] Suárez-Cuenca JA, Robledo-Nolasco R, Alcántara-Meléndez MA, Díaz-Hernandez LJ, Vera-Gómez E, Hernández-Patricio A, et al Coronary progenitor cells and soluble biomarkers in cardiovascular prognosis after coronary angioplasty. J Vis Exp 2020;155. 10.3791/60504 32065158

[26] Rigato M, Fadini GP. Circulating stem/progenitor cells as prognostic biomarkers in macro- and microvascular disease: a narrative review of prospective observational studies. Curr Med Chem 2018;25:4507–17. 10.2174/0929867324666170920154020 28933297

[27] Mizuno T, Hoshino T, Ishizuka K, Toi S, Takahashi S, Wako S, et al Association of circulating CD34 + cells level and prognosis after ischemic stroke. Int J Stroke 2024;19:460–9. 10.1177/17474930231217192 37978860

[28] Ye G, Chen X, Zhou Y, Zhou J, Song Y, Yang X, et al Prognostic value of endothelial progenitor cells in acute myocardial infarction patients. Mediators Inflamm 2023;2023:4450772. 10.1155/2023/4450772 37899988 PMC10613116

[29] Bakbak E, Verma S, Krishnaraj A, Quan A, Wang C-H, Pan Y, et al Empagliflozin improves circulating vascular regenerative cell content in people without diabetes with risk factors for adverse cardiac remodeling. Am J Physiol Heart Circ Physiol 2023;325:H1210–22. 10.1152/ajpheart.00141.2023 37773589

[30] Capoccia BJ, Robson DL, Levac KD, Maxwell DJ, Hohm SA, Neelamkavil MJ, et al Revascularization of ischemic limbs after transplantation of human bone marrow cells with high aldehyde dehydrogenase activity. Blood 2009;113:5340–51. 10.1182/blood-2008-04-154567 19324906 PMC2686196

[31] Putman DM, Cooper TT, Sherman SE, Seneviratne AK, Hewitt M, Bell GI, et al Expansion of umbilical cord blood aldehyde dehydrogenase expressing cells generates myeloid progenitor cells that stimulate limb revascularization. Stem Cells Transl Med 2017;6:1607. 10.1002/sctm.16-0472 28618138 PMC5689765

[32] Putman DM, Liu KY, Broughton HC, Bell GI, Hess DA. Umbilical cord blood-derived aldehyde dehydrogenase-expressing progenitor cells promote recovery from acute ischemic injury. Stem Cells 2012;30:2248–60. 10.1002/stem.1206 22899443

[33] Cooper TT, Sherman SE, Kuljanin M, Bell GI, Lajoie GA, Hess DA. Inhibition of aldehyde dehydrogenase-activity expands multipotent myeloid progenitor cells with vascular regenerative function. Stem Cells 2018;36:723–36. 10.1002/stem.2790 29377410

[34] Terenzi DC, Bakbak E, Trac JZ, Al-Omran M, Quan A, Teoh H, et al Isolation and characterization of circulating pro-vascular progenitor cell subsets from human whole blood samples. STAR Protoc 2021:2:100311. 10.1016/j.xpro.2021.100311 33554145 PMC7856468

[35] Yoder MC, Mead LE, Prater D, Krier TR, Mroueh KN, Li F, et al Redefining endothelial progenitor cells via clonal analysis and hematopoietic stem/progenitor cell principals. Blood 2006;109:1801–9. 10.1182/blood-2006-08-043471 17053059 PMC1801067

[36] Rogacev KS, Seiler S, Zawada AM, Reichart B, Herath E, Roth D, et al CD14++CD16 + monocytes and cardiovascular outcome in patients with chronic kidney disease. Eur Heart J 2011;32:84–92. 10.1093/eurheartj/ehq371 20943670

[37] Krychtiuk KA, Lenz M, Richter B, Hohensinner PJ, Kastl SP, Mangold A, et al Monocyte subsets predict mortality after cardiac arrest. J Leukoc Biol 2021;109:1139–1146. 10.1002/JLB.5A0420-231RR. 33020969 PMC8247267

[38] Kristiansen M, Graversen JH, Jacobsen C, Sonne O, Hoffman H-J, Law SKA, et al Identification of the haemoglobin scavenger receptor. Nature 2001;409:198–201. 10.1038/35051594 11196644

[39] Paradis P, Schiffrin EL. CXCL1–CXCR2 lead monocytes to the heart of the matter. Eur Heart J 2018;39:1832–4. 10.1093/eurheartj/ehy114 29528395 PMC5963305

[40] Hofbauer TM, Ondracek AS, Mangold M, Scherz T, Nechvile J, Seidl V, et al Neutrophil extracellular traps induce MCP-1 at the culprit site in ST-segment elevation myocardial infarction. Front Cell Dev Biol 2020;8:564169. 10.3389/fcell.2020.564169. 33240874 PMC7680894

[41] Zong P, Feng J, Yue Z, Yu AS, Vacher J, Jellison ER, et al TRPM2 deficiency in mice protects against atherosclerosis by inhibiting TRPM2–CD36 inflammatory axis in macrophages. Nat Cardiovasc Res 2022;1:344–60. 10.1038/s44161-022-00027-7 35445217 PMC9015693

[42] Lutgens E, Atzler D, Döring Y, Duchene J, Steffens S, Weber C. Immunotherapy for cardiovascular disease. Eur Heart J 2019;40:3937–46. 10.1093/eurheartj/ehz283 31121017

[43] Wang L, Zhang Y-L, Lin Q-Y, Liu Y, Guan X-M, Ma X-L, et al CXCL1–CXCR2 axis mediates angiotensin II-induced cardiac hypertrophy and remodelling through regulation of monocyte infiltration. Eur Heart J 2018;39:1818–31. 10.1093/eurheartj/ehy085 29514257

[44] Hoyer FF, Nahrendorf M. Neutrophil contributions to ischaemic heart disease. Eur Heart J 2017;38:465–72. 10.1093/eurheartj/ehx017 28363210

[45] Soehnlein O . Multiple roles for neutrophils in atherosclerosis. Circ Res 2012;110:875–88. 10.1161/CIRCRESAHA.111.257535 22427325

[46] Cappellari R, D’Anna M, Menegazzo L, Bonora BM, Albiero M, Avogaro A, et al Diabetes mellitus impairs circulating proangiogenic granulocytes. Diabetologia 2020;63:1872–84. 10.1007/s00125-020-05142-3 32306097

[47] Hess DA, Craft TP, Wirthlin L, Hohm S, Zhou P, Eades WC, et al Widespread nonhematopoietic tissue distribution by transplanted human progenitor cells with high aldehyde dehydrogenase activity. Stem Cells 2008;26:611–20. 10.1634/stemcells.2007-0429 18055447 PMC3045698

[48] Hess DA, Meyerrose TE, Wirthlin L, Craft TP, Herrbrich PE, Creer MH, et al Functional characterization of highly purified human hematopoietic repopulating cells isolated according to aldehyde dehydrogenase activity. Blood 2004;104:1648–55. 10.1182/blood-2004-02-0448 15178579

[49] Xie D, Li Y, Xu M, Zhao X, Chen M. Effects of dulaglutide on endothelial progenitor cells and arterial elasticity in patients with type 2 diabetes mellitus. Cardiovasc Diabetol 2022;21:1–14. 10.1186/s12933-022-01634-1 36199064 PMC9533545

[50] Bonaca MP, Catarig A-M, Houlind K, Ludvik B, Nordanstig J, Ramesh CK, et al Semaglutide and walking capacity in people with symptomatic peripheral artery disease and type 2 diabetes (STRIDE): a phase 3b, double-blind, randomised, placebo-controlled trial. Lancet 2025;405:1580–93. 10.1016/S0140-6736(25)00509-4 40169145

[51] Wong CK, McLean BA, Baggio LL, Koehler JA, Hammoud R, Rittig N. Central glucagon-like peptide 1 receptor activation inhibits toll-like receptor agonist-induced inflammation. Cell Metab 2024;36:130–43. 10.1016/j.cmet.2023.11.009 38113888

[52] Sforza A, Vigorelli V, Rurali E, Perrucci GL, Gambini E, Arici M, et al Liraglutide preserves CD34 + stem cells from dysfunction induced by high glucose exposure. Cardiovasc Diabetol 2022;21:1–16. 10.1186/s12933-022-01486-9 35397526 PMC8994898

[53] Chan JSF, Ussher JR. Vascular regeneration: a new mechanism of glucagon-like peptide-1 receptor agonist-mediated cardioprotection? Am J Physiol Heart Circ Physiol 2024;327:H406–8. 10.1152/ajpheart.00446.2024 38995213

[54] Wilding JPH, Batterham RL, Calanna S, Davies M, Van Gaal LF, Lingvay I, et al Once-weekly semaglutide in adults with overweight or obesity. N Engl J Med 2021;384:989–1002. 10.1056/NEJMoa2032183 33567185

[55] McGowan BM, Bruun JM, Capehorn M, Pedersen SD, Pietiläinen KH, Muniraju HAK, et al Efficacy and safety of once-weekly semaglutide 2·4 mg versus placebo in people with obesity and prediabetes (STEP 10): a randomised, double-blind, placebo-controlled, multicentre phase 3 trial. Lancet Diabetes Endocrinol 2024;12:631–42. 10.1016/S2213-8587(24)00182-7 39089293

[56] Davies M, Færch L, Jeppesen OK, Pakseresht A, Pedersen SD, Perreault L, et al Semaglutide 2·4 mg once a week in adults with overweight or obesity, and type 2 diabetes (STEP 2): a randomised, double-blind, double-dummy, placebo-controlled, phase 3 trial. Lancet 2021;397:971–84. 10.1016/S0140-6736(21)00213-0 33667417

[57] Laferrère B, Heshka S, Wang K, Khan Y, McGinty J, Teixeira J, et al Incretin levels and effect are markedly enhanced 1 month after roux-en-Y gastric bypass surgery in obese patients with type 2 diabetes. Diabetes Care 2007;30:1709–16. 10.2337/dc06-1549 17416796 PMC2743330

[58] Husain M, Birkenfeld AL, Donsmark M, Dungan K, Eliaschewitz FG, Franco DR, et al Oral semaglutide and cardiovascular outcomes in patients with type 2 diabetes. N Engl J Med 2019;381:841–51. 10.1056/NEJMoa1901118 31185157

