# Supplementary material for: Semaglutide promotes bone marrow–derived progenitor cell flux towards an anti-inflammatory and pro-regenerative profile in high-risk patients: the SEMA-VR CardioLink-15 trial
Source: Eur Heart J. 2025 Aug 31;47(10):1171–82. doi: 10.1093/eurheartj/ehaf690 (PMC13016833; doi:10.1093/eurheartj/ehaf690)
Supplement: ehaf690_Supplementary_Data [file ehaf690_supplementary_data.docx]

**Table S1. Key resources used for VR progenitor cell enumeration during the SEMA-VR CardioLink-15 trial**

| Reagent or Resource | Source | Identifier |
| --- | --- | --- |
| Antibodies | | |
| Anti-human CD34 (APC) | BioLegend | Cat# 343608 |
| Anti-human CD133 (BV421) | BioLegend | Cat# 393908 |
| Anti-human CD45 (PE-Cy7) | BioLegend | Cat# 368532 |
| Anti-human CD14 (APC) | BioLegend | Cat# 399206 |
| Anti-human CD36 (BV421) | BioLegend | Cat# 336230 |
| Anti-human CD163 (PE-Cy7) | BioLegend | Cat# 333614 |
| Anti-human CD86 (PE-Dazzle 594) | BioLegend | Cat# 374218 |
| Anti-human CD192 (PE) | BioLegend | Cat# 357206 |
| Anti-human CD66b (APC) | BioLegend | Cat# 305118 |
| Anti-human CXCR2 (PE-Dazzle 594) | BioLegend | Cat# 320722 |
| Anti-human CD49d (PE) | BioLegend | Cat# 304304 |
| Human TruStain FcX^TM^ | BioLegend | Cat# 422302 |
| Chemicals, peptides, and recombinant proteins | | |
| Fetal bovine serum | Sigma-Aldrich | Cat# F2442 |
| Ammonium chloride lysis buffer | STEMCELL Technologies | Cat# 07850 |
| Phosphate buffered saline | Gibco | Cat# 10010023 |
| Commercial assays | | |
| Aldefluor^TM^ kit | STEMCELL Technologies | Cat# 01700 |
| CellROX^TM^ Deep Red kit | Invitrogen^TM^ | Cat# C10422 |
| Software | | |
| FlowJo^TM^ Software | BD Biosciences | Version 10.8.2 <https://www.flowjo.com/> |
| GraphPad Prism | GraphPad Software | Version 10.1.1 <https://www.graphpad.com/> |
| Other | | |
| SepMate^TM^ PBMC isolation tubes | STEMCELL Technologies | Cat# 85415 |
| Vacutainer EDTA Tubes | BD Biosciences | Cat# 366643 |
| Vacutainer SST^TM^ Tubes | BD Biosciences | Cat# 367986 |
| CytoFLEX LX Flow Cytometer | Beckman Coulter | N/A |

**Table S2. Effect of semaglutide on the percent change from baseline in circulating ALDH^hi^SSC^low^ VR progenitor cells and ALDH^hi^SSC^hi^ pro-inflammatory granulocyte precursors.**

|  | Usual Care (*n* = 24) | Semaglutide (*n* = 22) | *P* |
| --- | --- | --- | --- |
| ALDH^hi^SSC^low^ | +0.8% | +34.8% | **0.036** |
| CD34^+^ | +10.5% | +27.7% | 0.469 |
| CD133^+^ | +36.4% | +41.0% | 0.887 |
| CD45^+^ | -2.8% | +40.1% | **0.017** |
| CD34^+^CD133^+^ | +50.6% | +25.6% | 0.492 |
| CD34^+^CD133^+^CD45^-^ | -2.3% | +66.2% | **0.037** |
| ALDH^hi^SSC^mid^ | +13.4% | +18.8% | 0.862 |
| CD14^+^ | +19.8% | +25.2% | 0.879 |
| CD14^+^CCR2^+^ | +20.2% | +25.1% | 0.889 |
| CD14^+^CD86^+^ | +17.4% | +24.9% | 0.828 |
| CD14^+^CD163^+^ | +25.7% | +36.3% | 0.801 |
| CD14^+^CD36^+^ | +18.9% | +27.1% | 0.818 |
| ALDH^hi^SSC^hi^ | +0.3% | -50.8% | **0.002** |
| CD49d^+^ | +30.4% | -32.7% | **0.021** |
| CD66b^+^ | +21.1% | -53.6% | **0.007** |
| CXCR2^+^ | +30.8% | -56.7% | **<0.001** |

Data are presented as mean percent change from baseline (%) in the absolute number of VR cell subsets. ALDH, aldehyde dehydrogenase; SSC, side scatter. Paired t-tests were performed between the percent change values from the usual care (*n* = 24) vs. semaglutide (*n* = 22) groups.

**
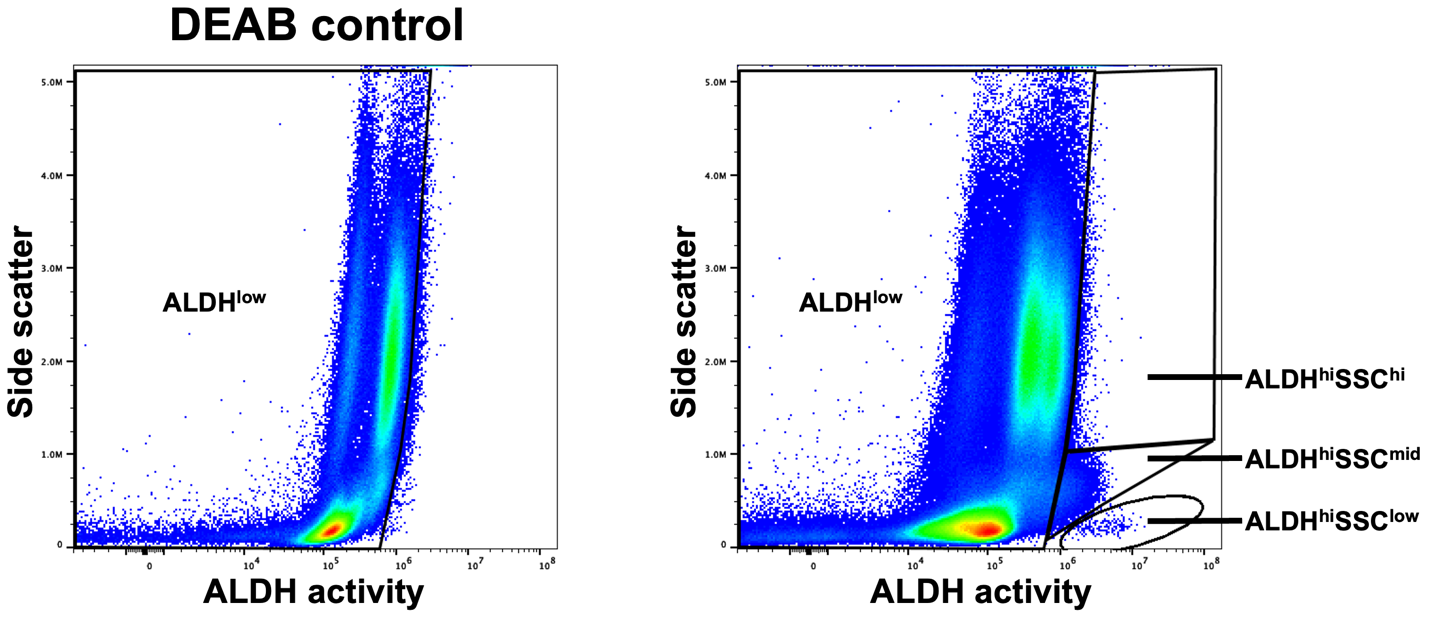
**

**Figure S1. Representative flow cytometry plots illustrating the gating strategy for ALDH^hi^ vs. ALDH^low^ cell populations.** Aldehyde dehydrogenase (ALDH) activity was assessed using a fluorescent substrate in the presence or absence of N,N-diethylaminobenzaldehyde (DEAB), a selective ALDH inhibitor used to define background fluorescence. DEAB gating enabled the delineation of three ALDH^hi^ subpopulations based on side scatter characteristics: primitive VR progenitor cells (ALDH^hi^SSC^low^), monocytes (ALDH^hi^SSC^mid^), and granulocyte precursors (ALDH^hi^SSC^hi^).

ALDH, aldehyde dehydrogenase; DEAB, N,N-diethylaminobenzaldehyde; SSC, side scatter.

**
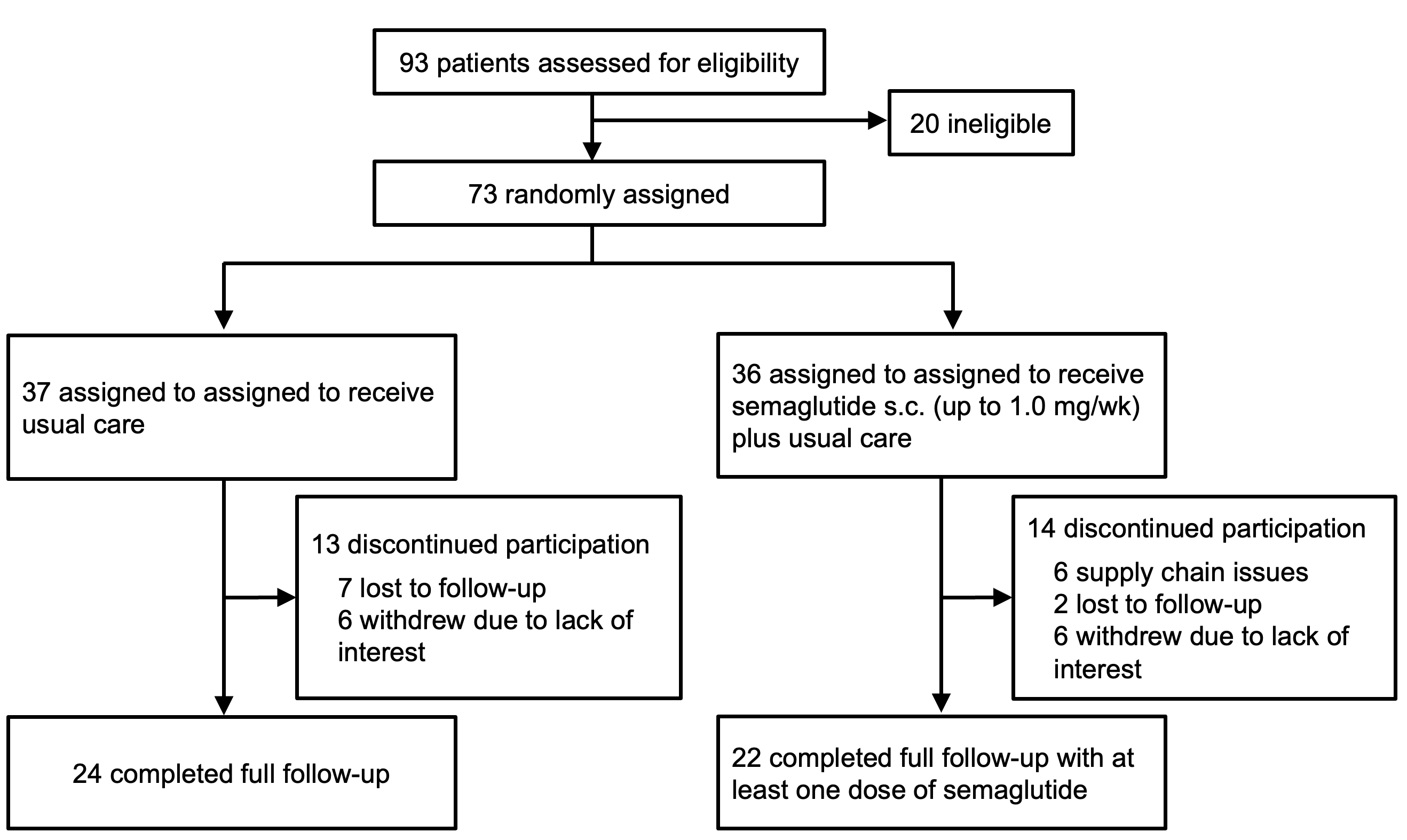
**

**Figure S2. CONSORT diagram flow of participants through the SEMA-VR CardioLink-15** **clinical trial**


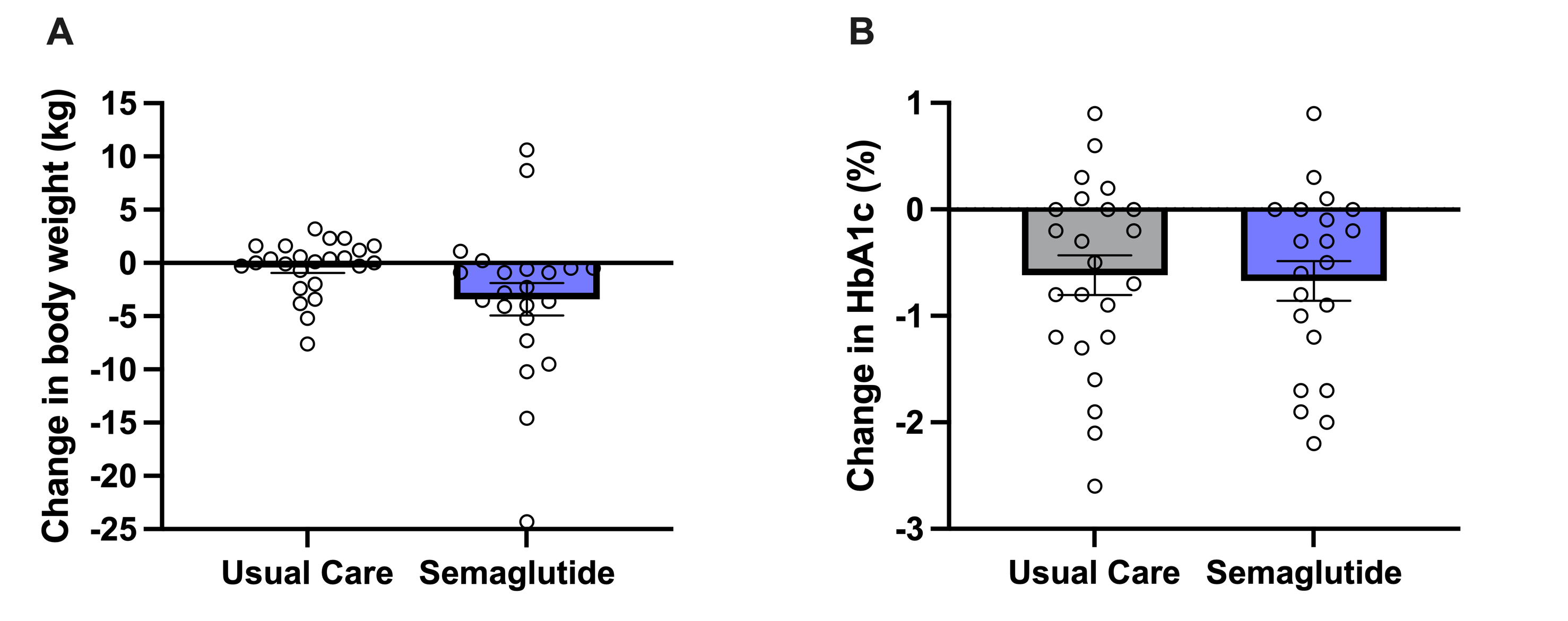


**Figure S3. Change in body weight and glycated hemoglobin in individuals receiving usual care vs. semaglutide for 6 months.** Mean changes in (A) body weight and (B) glycated hemoglobin (HbA1c) were assessed at 6-month follow-up in participants receiving usual care (*n* = 24) compared with those assigned to subcutaneous semaglutide (*n* = 22). No statistically significant differences were observed between groups in either parameter over the treatment period.
